# Medium and long-term complications difference between laparoscopic transcystic common bile duct exploration versus endoscopic sphincterotomy against choledocholithiasis: A protocol for systematic review and meta-analysis

**DOI:** 10.1097/MD.0000000000024104

**Published:** 2021-01-22

**Authors:** Quanxin Liu, Tao Li, Zhangdong Feng, Wei Han

**Affiliations:** a Beijing Luhe Hospital, Capital Medical University, Tongzhou, Beijing; b The Second Hospital of Hebei Medical University, Shijiazhuang, Hebei province, China.

**Keywords:** common bile duct stone, complications, endoscopic sphincterotomy, laparoscopic

## Abstract

**Background:**

Common bile duct stone (CBDS) is typically manifested with abdominal pain, chills, fever, and jaundice. Laparoscopic transcystic common bile duct exploration (LTCBDE) and endoscopic sphincterotomy (EST) are currently the main minimally invasive methods for the treatment of CBDS. However, there are few studies about the differences of medium and long-term complication after EST or LTCBDE. Therefore, we will conduct a meta-analysis and systematic review to systematically evaluate the difference of medium and long-term complications between EST and LTCBDE against CBDS.

**Methods:**

Randomized controlled trials of EST or LTCBDE against CBDS will be searched in several English and Chinese databases with the following vocabularies: “laparoscopic transcystic common bile duct exploration,” “endoscopic sphincterotomy,” “choledocholithiasis,” “common bile duct stone” until December, 2020. Two reviewers will independently conduct the literature extraction, risk of bias assessment, and statistical analysis.

**Results and Conclusions:**

The study will help to systematically evaluate the difference of medium and long-term complication between EST and LTCBDE against CBDS.

**OSF Registration number:**

DOI 10.17605/OSF.IO/5U7SA.

## 1 Introduction

Choledocholithiasis, also known as common bile duct stone (CBDS), is the presence of pigment stones or mixed stones at the lower common bile duct (CBD).^[1,2]^ In recent years, the incidence of CBDS has gradually increased and is found in approximately 10% to 15% of patients with cholelithiasis.^[3]^ The typical clinical manifestations of CBDS are abdominal pain, chills, fever, and jaundice. It will further aggravate the infection to result in purulent obstructive cholangitis with toxic shock manifestations, such as high fever, irritability, and lethargy if not timely cured.

The optimal treatment of CBDS involves choledocholithotomy, which is the removal of the gallstone from the bile duct. The conventional operation, open choledocholithotomy, could effectively relieve the symptoms, but it may cause a large trauma with a slow recovery time. Nowadays, since its introduction, laparoscopic surgery^[4]^ has become the primary treatment for patients with CBDS with the advantages of smaller trauma and shorter recovery time.^[5,6]^ Laparoscopic transcystic common bile duct exploration (LTCBDE) and endoscopic sphincterotomy are currently the main minimally invasive methods for the treatment of CBDS. During the process of EST, the muscle between the CBD and the pancreatic duct will be cut and a catheter will be used to remove gallstones. There is no surgical incision on the abdominal wall, and the operation is simple and short in time. However, EST is also associated with long-term complications in 8% to 10% of patients, including recurrent ductal stones, cholangitis, and papilla stenosis.^[7,8]^

LTCBDE is a procedure for exploring and removing stones via the transcystic route. It could maintain the integrity of the CBD, and avoid the recurrence induced by the changes of biliary tract hydromechanics.^[9–11]^ LTCBDE has become a widely accepted method against patients with CBDS and suspected CBDS, with the success rate over 90% and a lower long-term complication rate.^[12,13]^ Both EST and LTCBDE could effectively treat CBDS with high success rates.^[14,15]^ However, there are few studies about the differences of medium and long-term complications after EST or LTCBDE. Therefore, we will conduct a meta-analysis and systematic review to systematically evaluate the difference of medium and long-term complications between EST and LTCBDE against CBDS.

## 2 Methods

### 2.1 Study registration

This protocol has been registered on open science framework (OSF) (Registration number: DOI 10.17605/OSF.IO/5U7SA). And it is drafted under the guidance of the preferred reporting items for systematic reviews and meta-analyses protocols (PRISMA-P).^[16]^

### 2.2 Ethics

The data will be derived from previously published studies, and ethical approval is not required.

### 2.3 Inclusion criteria

#### 2.3.1 Type of studies

We will include randomized controlled trials (RCTs) studies of LTCBDE or EST against CBDS in Chinese and English.

#### 2.3.2 Type of participants

Participants with CBDS, in spite of nationality, race, age, and gender.

#### 2.3.3 Type of interventions

RCTs of LTCBDE or EST against CBDS.

#### 2.3.4 Type of outcome measures

Outcomes include success rate, operative time, intraoperative laparotomy rate, postoperative length of hospital stay, recurrence, and complications (abdominal pain, fever, jaundice, vomiting, diarrhea, cholangitis, and pancreatitis).

### 2.4 Exclusion criteria

1. Studies without appropriate randomization;
2. Studies without completely described outcomes;
3. Duplicated published literatures;
4. Studies with data unable to retrieve;

### 2.5 Search strategy

Studies will be searched in Cochrane Library, Medline, PubMed, Embase, Chinese Biological and Medical database (CMB), China National Knowledge Infrastructure (CNKI), Chongqing VIP, and Wanfang until Dec, 2020 with the following vocabularies: “laparoscopic transcystic common bile duct exploration,” “endoscopic sphincterotomy,” “Choledocholithiasis,” “common bile duct stone,” as listed in Table 1.

**Table 1:** Search strategy in PubMed database.

Number	Search terms
#1	Common bile duct stone [Title/Abstract]
#2	Choledocholithiasis [Title/Abstract]
#3	Common Bile Duct Calculi [Title/Abstract]
#4	Common Bile Duct Gallstones [Title/Abstract]
#5	Gall Stones, Common Bile Duct [Title/Abstract]
#6	Common Bile Duct Calculi [MeSH Terms]
#7	#1 OR #2 OR #3 OR #4 OR #5 OR #6
#8	Laparoscopic transcystic common bile duct exploration [Title/Abstract]
#9	LTCBDE [Title/Abstract]
#10	endoscopic sphincterotomy [Title/Abstract]
#11	#8 OR #9 OR #10
#12	#7 AND #11

### 2.6 Data extraction

**Figure 1:**
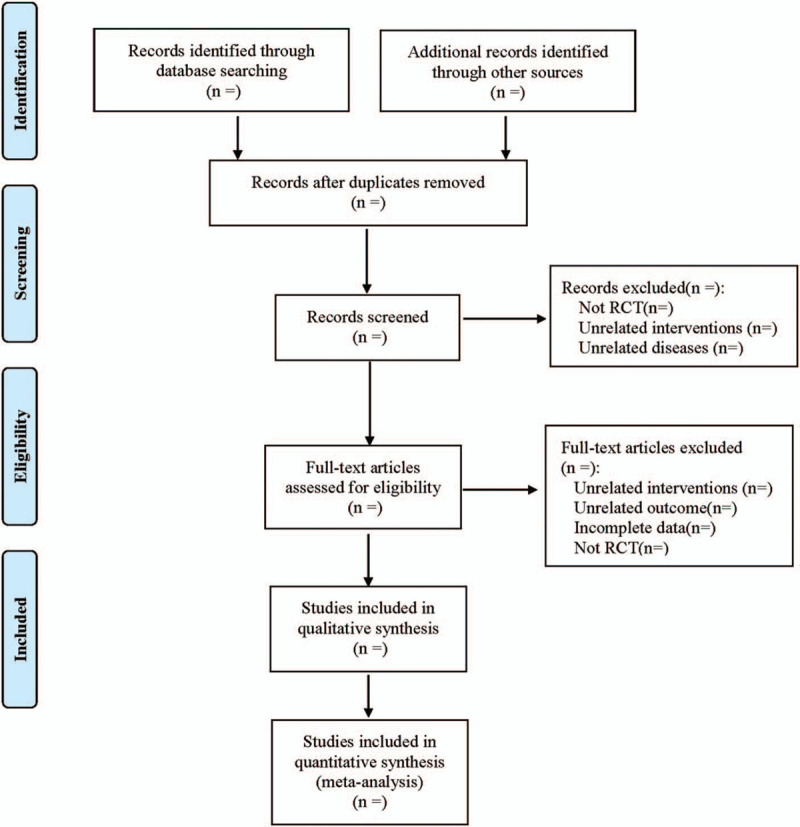
Flow diagram.

The literature screening process is performed by Endnote X7 (Fig. 1). Data extraction will be performed by 2 reviewers with Excel 2019, including title, authors, publication year, journal, language, age, races, gender, randomization, blind, concealment, interventions, outcomes, complications, and adverse events. The senior author will be consulted to decide for discrepancies.

### 2.7 Risk of bias assessment

Two reviewers will independently evaluate the risk of bias (random sequence generation, allocation concealment, blinding method, incomplete outcomes, selective report) according to the Cochrane Handbook of Systematic Reviewers. The qualities will be evaluated by the Newcastle-Ottawa Quality Assessment Scale (NOS).

### 2.8 Statistical analysis

#### 2.8.1 Data synthesis

RevMan 5.3 (Cochrane Collaboration, Oxford, United Kingdom) will be applied for analyzing statistics. The relative risk (RR) with the 95% confidence interval (CI) will be applied for dichotomous variables, and weighted mean difference (WMD) with 95% CI for continuous variables. Heterogeneity test is evaluated with Q test and quantified with I^2^ statistic. The random effect model will be used for analysis without obvious clinical/methodological heterogeneity.

#### 2.8.2 Dealing with missing data

The corresponding author will be contacted by any ways to get the missing or incomplete data.

#### 2.8.3 Sensitivity analysis

A one-by-one elimination method will be adopted for sensitivity analysis to test the stability of meta-analysis results of indicators.

#### 2.8.4 Reporting bias

If the included studies are more than 10, funnel plot will be used to qualitatively detect reporting bias by Egger and Begg tests.

#### 2.8.5 Evidence quality evaluation

Evidence quality will be rated in high, moderate, low, and very low by the Grading of Recommendations Assessment, Development, and Evaluation (GRADE) with bias risk, consistency, directness, precision, and publication bias.

## 3 Discussion

CBDS can be caused by repeated bile duct inflammatory, bile duct malformation, and bile stasis, or secondary stones caused by gallstones or intrahepatic bile duct stones discharged into the CBD. It can easily lead to obstruction of CBD and acute suppurative cholangitis, which can seriously cause septic shock to threaten the life and health of patients. With the development of minimally invasive instruments, laparoscopy and choledochoscope for CBDS has become the main means of contemporary surgeons. Minimally invasive treatment of CBDS has the advantages of safety, quick recovery, less pain, and less economic burden. LCBDE and EST^[17]^ are the main minimally invasive operation for the treatment of CBDS, and the curative effect is satisfactory. However, there is no consensus on the medium and long-term complication difference between the 2 operations. Therefore, in this study, we try to conduct a meta-analysis and systematic review to evaluate the medium and long-term complication differences between EST and LTCBDE against CBDS. Also, we need to address some limitations. First, we only include studies in Chinese and English and it may result in certain selective bias. Second, the number and quality of CBDS will affect the conclusions in different trials.

## Author contributions

**Data collection:** Quanxin Liu.

**Data curation:** Liu Quanxin.

**Funding acquisition:** Wei Han.

**Funding support:** Wei Han.

**Literature retrieval:** Tao Li and Zhangdong Feng.

**Software operating:** Tao Li and Zhangdong Feng.

**Software:** Tao Li, Zhangdong Feng.

**Supervision:** Zhangdong Feng.

**Writing – original draft:** Quanxin Liu and Tao Li.

**Writing – review & editing:** Quanxin Liu and Wei Han.
